# The role of gender inequities in women's access to reproductive health services: a population-level study of Simiyu Region Tanzania

**DOI:** 10.1186/s12889-023-15839-w

**Published:** 2023-06-09

**Authors:** Jane Tesha, Agatha Fabian, Serafina Mkuwa, Gaspery Misungwi, Frida Ngalesoni

**Affiliations:** 1 Amref Health Africa, Tanzania. Ali Hassan Mwinyi Road Plot 1019 P O, Box 2773, Dar es Salaam, Tanzania; 2 Department of Public Health, College of Health Sciences, Dodoma University, P.O.Box 395, Dodoma, Tanzania

**Keywords:** Women, Gender inequities, Reproductive health services, Simiyu

## Abstract

**Background:**

Amref Health Africa, with support from Global Affairs Canada, examines if women's access to reproductive health services in Tanzania is affected by Gender social norms, decision-making power, roles and responsibility, and access to resources in relation to the utilization of reproductive Health Services in Tanzania. A Gender Need Assessment (GNA) was conducted in five districts in ' 'Tanzania Simiyu Region to improve the infrastructure, supply, quality, and demand for integrated Reproductive, Maternal, Newborn, and Child and Adolescent Health (RMNCAH), Nutrition, and Water, Sanitation, and Hygiene (WASH) services. The analysis identifies gender as a fundamental maternal and child health driver through existing gender inequality at the household and community levels that dictates women's status.

**Methods:**

The qualitative assessment involved data collected from gender- and age-desegregated focus group discussions (FGDs) and in-depth interviews (IDIs) of key informants in three districts; Bariadi, Busega, and Meatu, in Simiyu region, Tanzania. Participants comprised 8-10 married women and men, unmarried women and men, and adolescent boys and girls. A total of 129 participants were involved in the FGDs.

**Results:**

This paper reports the critical drivers influencing gender inequality in Simiyu by detailing how Gender inequality affected women's access to reproductive health care in relation to; gender social norms, decision-making power, access to resources at the household and community level, roles and responsibilities, including men's and 'boys' roles are more valued than the roles and responsibilities of women and girls resulted to limited free time to do things for themselves, such as visiting the health facilities for RMNCAH.

**Conclusions:**

This paper explored gender-based enablers and/or barriers influencing women and girls' realization of their sexual and reproductive health and rights. It was found that social norms, decision-making powers, and lack of access to and control over resources presented as key barriers. In contrast, continuous community sensitization and increased scope of women's participation in decision-making served as enabling environment to overcome gender inequities that influence woman's use of RMNCAH services in Tanzania. Such insights will shape interventions geared towards valuing differences in a manner that overcome gender inequities that influence woman's use of RMNCAH services in Tanzania.

## Background

Globally approximately 536,000 women die from pregnancy-related causes every year, with Sub-Saharan Africa (SSA) counting almost 50% of these deaths [1]. Tanzania has made progress in maternal death reduction, estimated at 292/per 100,000 live births in 2020, from 556/per 100,000 births in 2016 [2]. However, despite all the efforts, maternal mortality remained high in Tanzania and SSA countries in general. These figures show that most SSA countries are not on the right track to achieving r the sustainable development goal (SDG 3) of less than 70 per 10000 by 2030 [3].

While in East Asia, the Pacific, and South and North America, 9 out of 10 births are attended by trained birth attendants. In SSA, just half of the births (46 %) are conducted by a skilled birth attendant (SBA) in health facilities [4]. As indicated by Helland et al., reproductive rights are human rights. The gap of women not attended by SBA can be considered a violation of their right to life and health that must be enjoyed equally based on sex, race, age, ethnicity, or other factors. [5]. Such violations brought attention to the United Nations to guide on using a human rights-based approach to maternal healthcare for all women. In addition, women's realization of their right to access skilled maternity services is a critical human rights strategy to secure service availability, accessibility, and acceptability [6]. The emphasis continues in women-oriented human rights provisions, such as article 14 of the Convention on the Elimination of all Forms of Discrimination Against Women (CEDAW) [7] and Article 14 of the African Women's Protocol (AWP) [8]. Therefore, an essential aspect of realizing women's rights is human rights is to ensure women's access and utilization of healthcare services during pregnancy and birth.

In many African societies, including Tanzania, men are considered breadwinners and expected to bring in income to support the family and provide funds for health care [9]. In Tanzania, maternal and child health outcomes are affected by such gender social norms. Simiyu region is one of the regions with mainly male predominant culture. The health outcomes and utilization of reproductive, maternal, and child health services amongst women are significantly affected by gender social norms, decision-making power, roles and responsibility, and access to resources [2].

This study, therefore, seeks to explore gender-based enablers and/or barriers that influence women and girls' realization of their sexual and reproductive health and rights in the Simiyu Region of Tanzania, which is faced with high maternal mortality rates. The results of this work will inform the Government of Tanzania and other development and implementing partners of tailor-made gender-responsive interventions to be incorporated into maternal and child health interventions to improve maternal and child health outcomes

## Methods

### Conceptual framework

**Fig. 1:**
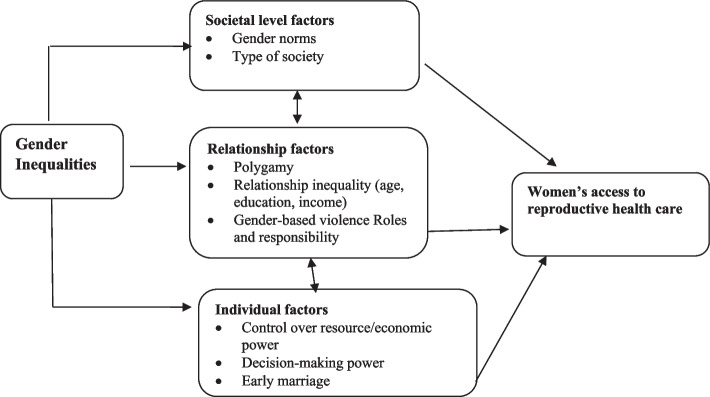
Conceptual framework [10]

This study employed a conceptual framework adapted from the ecological systems theory. This framework was used to analyze the linkage between women's access and utilization of reproductive health care and gender inequities at three different multidimensional levels of social, relationships, and individual factors (see Fig. 1 below).

### Study area

The study was conducted in the Simiyu Region, Tanzania, one of the 31 Regions of Tanzania. The region is in northwestern Tanzania, consisting of 2,140,497(48%M:52%F) [11].

### Study design and sampling

This was a cross-sectional qualitative study design. Data were collected through in-depth interviews (IDIs) of key informants and Focus Group Discussions (FGDs). A total of 16 FGDs were conducted, and each FGD comprised 8-10 participants married women and men, unmarried women and men, and adolescent boys and girls aged 15 to 19 years. According to the Tanzania marriage Act of 1971, the marriage age for girls in Tanzania is 15 years; hence the study considered adolescents aged 15 to 19 years, excluding the 10-14 years of age as per the WHO definition. The distribution of the sample of the respondents in the FGDs is provided in Table 1.

The study also conducted IDIs with key informants, including District Medical Officers (DMO), District gender focal persons, District WASH coordinators, traditional leaders, District Reproductive and Child Health Coordinators (DRCHCOs), influential people, youth leaders, Village Executive Officers (VEO). A total of 30 IDIs were conducted, as shown in Table 2.

Purposive and simple random sampling was used in selecting districts, wards, villages, and study participants through a multistage sampling technique. In the initial stage, three districts, namely Bariadi, Busega, and Meatu, were selected purposively based on the tribe representation and a mix of urban and rural settings. Of the three districts chosen, Bariadi has urban characteristics; Busega has a mixture of urban and rural, while Meatu has rural characteristics with five ethnic groups. Next, two wards were randomly selected from each selected district, obtaining a total of six. Again, from each selected ward, one village was determined using the same technique making a total of six villages. All households were eligible for the study from each of the six selected villages. With the help of local leaders, participants from the families were invited to participate in the FGD, which was conducted separately for males, females, boys, and girls. Participants who met the inclusion criteria but refused to participate and those who were sick or not mentally fit were excluded from the study. Again, household members who had resided in the study area for less than six months were excluded because they would not have had enough local experience on gender issues.

### Data collection tools and methods

FGDs and IDI methods were used to collect data, and the tools used were the discussion guide for FGD and an interview guide for IDI. The two teams each comprised a researcher (moderator) and a research assistant (RAs) with experience in qualitative research involved in data collection. Both RAs underwent training for two days before data collection to familiarize themselves with data collection tools. All FGDs were recorded**.** During the data collection, the researchers moderated the FGDs and operated the tape recorder while an R.A. took notes. Data collection tools were pretested in one village, which was not included in the study. The modification of tools was made according to early experience and participant information. The interviews were conducted in a quiet place in the village/ward office or under a tree. The average time for FGDs and IDIs was between 60 and 90 minutes. Data were collected from May to June 2021.

### Data analysis

Before data analysis, the tape-recorded FGD and IDI were transcribed verbatim in Kiswahili. Then the research team translated the transcript from Kiswahili to English and compared it with the hand-written field notes. Nvivo software was used for organizing, storing, and retrieving data. The thematic content analysis approach was used in the analysis process, and four thematic areas emerged, namely gender social norms: decision-making, roles and responsibility, and access to resources in relation to the utilization of reproductive health services.

## Results

### Demographic distribution of study participants

One hundred and twenty-nine participants were involved in the FGDs with different categories and age groups. The majority of participants (38) were aged between 20-29 years, and most of them (36) were married men (See Table 1 below).

**Table 1:** Distribution of the FGDs participants by age, sex, and marital status

***Category***	***Age group (Years)***					
	***15-19***	***20-29***	***30-39***	***40-49***	***50-60***	***TOTAL***
*Married women*	*4*	*10*	*7*	*9*		*30*
*Married men*	*0*	*11*	*12*	*11*	*2*	*36*
*Un Married men*	*0*	*16*	*5*	*4*	*1*	*26*
*Unmarried women*	*0*	*0*	*3*	*0*	*0*	*3*
*Boys*	*16*	*0*	*0*	*0*	*0*	*16*
*Girls*	*17*	*1*	*0*	*0*	*0*	*18*
***TOTAL***	***37***	***38***	***27***	***24***	***3***	***129***

### Characteristics of the IDIs participants by age and sex

A total of 30 IDI was conducted with key informants, including 21 males and nine females. The majority (11) of study participants were aged between 30-39 years, as shown in Table 2 below

**Table 2:** Distribution of the IDIs participants by age and sex

***Age group***	***Male***	***Female***	***Total***
*20-29*	*1*	*0*	*1*
*30-39*	*6*	*5*	*11*
*40-49*	*6*	*3*	*9*
*50-60*	*6*	*1*	*7*
*'Didn't specify*	*2*	*0*	*2*
***Total***	***21***	***9***	***30***

### Gender social norms in relation to utilization of reproductive health services

FGDs participants were asked to share their experiences on the existing gender norms in their communities which may hinder women from utilizing reproductive health services. The majority mentioned social norms as the main drivers of gender inequalities among women, men, boys, and girls in the community. Most of them negatively perceive family planning services and cannot allow their spouses to use them. As a result, some women secretly opt for family planning (F.P.) methods without their spouse's knowledge. If uncovered, that could result in divorce or physical and psychological violence, limiting women from effectively utilizing maternal health services. For example, a participant provided a scenario as follows;

“*A woman who decided to use an implant family panning method secretly was forced to remove the plaster before arriving home for fear of being noticed by her husband. (IDI- District official, Meatu DC)*

Participants shared their experiences, emphasizing that every community has its traditions and directives. One of the community health workers said that sometimes they take severely malnourished children to the health facility, but they will be warned by the father to bring them home (mother and child) because he doesn't want her to be admitted. She further said that anemia is prevalent among pregnant women in Simiyu, which could be due to forbidding women to take some foods, including some fruits like pawpaw, which are crucial during pregnancy. This was narrated as follows;

*“Sometimes community health workers bring severely malnourished children to the health facility, but she/he will be warned by the father to bring them home (mother and child), no admission please." (A CHW - Meatu DC)*

For example, a traditional practice of paying dowry when the girls get married was also among the social norms of enslaved people, causing women and girls to be treated as enslaved people and commodities in the family, becoming inferior to men and limiting their access to reproductive services. This was elaborated as follows;

"*Our community believes in the tradition of having many kids, as we pay high bride prices, so they cannot give birth to a few children. For example, payment of 20 cows for bride price and the woman want to have only two children, it is not accepted, so we can't allow them to use family planning” (Married man - Busega DC)*

It was also observed that traditional social norms and practices would continue to exist in the community because after getting married, men stay in their father's households for up to 45 or 50 years. As a result, children will observe their father's brutal behavior and continue living under the same behavior when they grow up. Hence the limited access to RMNCAH services may persist in the study area if not intervened.

"*The traditional behaviors of men leading their family as they wish have been inherited from their forefathers. Anything said by a man should be obeyed; the woman cannot tell his man anything, and if a man listens to his wife, the members of the community will think that the man is the married one” (CHW - Busega DC*)

### Decision-making power in relation to access to reproductive health services

Men across all ethnic groups in the region dominate decision-making processes. This includes deciding on whether the RMNCAH services should be sought or not. Participants further reported that decision-making being solely at men's hands has been fatal. For example, it was noted that one woman died following an ectopic complication because her husband did not consent to the operation as he was away on safari.

It was also reported that women and girls and men and boys are valued differently across all ethnic groups that were interviewed. Men are considered to be superior in all tribes in Simiyu. Boys are preferred over girls, and this practice perpetuates gender inequality in almost all tribes interviewed. Women and girls are considered weak and inferior beings who cannot be trusted with anything.

This was elaborated as follows;

"We men, when you bring (marry) a woman, you have bought her, which means, if I bought her, she must bring me a profit. A woman is a housewife, a man is a decision-maker, and her job is to give birth to children for me to have a big family. You know a man will get hurt if a woman gets an education. If a woman gets money, she is cruel to her husband (Married man –Bariadi DC)

### Roles and responsibility in relation to access to reproductive health services

In Sukuma society the roles of men and women in the society are mainly patriarchal, where women are under the control of men hence leading to gender inequality. The findings from FGD revealed that in the Sukuma community, women and girls are responsible for all domestic duties ranging from cooking, collecting water and wood, taking care of patients, serving men, and sometimes farm activities, including animal grazing. They perform these activities with the assistance of children perpetuating men wanting many children to help perform family activities. This practice could limit women from utilizing maternal health services due to many responsibilities and limited time. This was reported as follows;

*"A woman is a housewife; her job is to give birth to many children to have a big family” (Community leader - Busega DC)*

It was also reported that some men misuse the family resources from farm activities due to alcoholic behavior. This may cause women to have limited financial resources, which could help in accessing maternal health care even when they have contributed to the financial gains of the family. One Sukuma female respondent reported as follows;

*“You know for Sukuma, women do almost everything; even if you want to access services, it becomes difficult because we don’t have time. For example, we have to wait and cook for the young boys who went for cattle grazing”. (Married Women - Busega district in Nyashimo village)*

On a positive note, some participants reported improvements regarding gender roles and responsibilities in the community owing to years of awareness created by the government and other health stakeholders. Participants said that, unlike in the past when women were seen as laborers working on the farmsteads, kitchen, and other household chores, men nowadays assist them by doing household activities like fetching water and wood, carrying luggage, and bringing food home. They also explained women's role in economic activities, as now they can engage in entrepreneurship and other economic activities. This helps them earn and increase their confidence in the household, including using reproductive and child health services. This was narrated as follows;

"This issue was alarming but has subsided after seminars and mothers joining entrepreneurial groups. Entrepreneurial groups have greatly contributed to mothers engaging in small businesses and stopping relying on men. When a man goes out for a drink, a woman goes to her business and feeds the family. A man lacks the strength to attack a woman because he knows a woman can live independent lives." (FGD with Community leader –Bariadi DC**)**

### Access to resources in relation to reproductive health services

In Sukuma societies, a traditional conception of access to resources and economic power is determined by the patriarchal system in which men hold primary power and predominate in social and economic roles at the family level, thus creating economic inequality between men and women. Consequently, lack of access to and control over resources affects women’s health-seeking behavior.

Economic empowerment is a pre-condition for realizing the full spectrum of human rights. Across all ethnic groups in the Simiyu region, women and girls do not have equal access to or control over resources such as land, livestock, money, houses, education, etc. Men, however, have access to and control over family resources and decide how and when the resources will be used. These practices impact women's access to quality RMNCAH services. Women do not have the confidence or financial autonomy to exert control over their lives, including the sexual and reproductive health aspects of life. For example, one of the DRHCOs shared a scenario below

"A woman in her 8th pregnancy took her husband to the health facility for counseling. She felt too tired to continue bearing children and asked for family planning. Her husband responded, “My mother had 13 of us; my target is 14 children” (CHW-Busega DC)

During FGDs, participants said that boys are given preference, access to family resources, are educated, and are provided with the best meals. Regarding inheritance, boys are given valuable resources like land, houses, and even cars. On the other hand, girls are given a share of household items like cooking pans, plates, clothes, etc. In some families where girls were given a share of livestock, they got less than boys. For example, one of the adolescent girls gave her a scenario that;

"In our family, we are five girls and one boy owning ten cows, when parents distributed these cows to us - a boy was given five cows while girls were given one cow each." (A girl, in FGD –Busega DC)

However, other participants reported that economic power or ownership of resources gives a person power over decision-making. It was also reported that when a woman is the owner of the properties, she may be disrespectful to her husband, or her respect for the husband becomes very low. . This notion was perceived as gender inequity that affects men and the other way around was not viewed as such as demonstrated by this quote from a community leader.

"If I can say from my side, I could say if the husband has economic power, it is unusual to observe gender inequalities in the family. However, in a case where the wife owns most or all of the family properties and the husband does not own, a woman will not respect her husband as ahead of the family “Community leader - Bariadi DC)

## Discussion

The results presented a portrait of how gender inequities may affect reproductive health services access and utilization in Tanzania within the study region. In general, it was found that access and utilization are influenced by the intersection of access to resources, division of labour, social norms, and decision-making, which institute gendered power relations. In addition, the Gender Need Assessment (GNA) conducted in Simiyu [12] revealed that gender inequity has limited women's ability to access maternal healthcare services and utilization in multiple ways. The GNA explored gender-based roles and responsibilities, social norms, decision-making power, and access to and control over resources.

### Gender social norms in relation to utilization of reproductive health services

This study showed that as power holders in the household, men decide when women should access reproductive health services. Subsequently, women are not treated as individuals in their own right to access reproductive health services due to the harmful practices resulting from social conventions and social norms. This finding is consistent with one study from Ghana, which indicated that women face difficulty accessing reproductive health services because they often lack the independence to make decisions even when seeking care [4].

In FGDs and individual interviews, it was reported that men and boys were valued differently than women and girls and considered weak and inferior across all ethnic groups, unlike men who considered themselves more powerful and decided over women. This practice perpetuates gender inequalities in the community, leaving women valueless and voiceless. Studies have indicated how certain sociocultural norms and values affect women's autonomy in making decisions about their health [13]. Consequently, many women still seek the permission of their husbands or a partner even in health-related emergencies; as a result, some have experienced childbirth-related complications [14].

Likewise, the division of labor at households is shaped by social norms, where women are more likely to handle housework and childcare activities. A similar study in rural Gambia confirmed this finding, reporting that rural women were still expected to endure heavy workloads, which are unpaid care work, while pregnant that had limited opportunities and resources to access ANC. Moreover, strenuous activities during pregnancy can result in adverse health outcomes for women and their unborn children. Pregnant women who engaged in strenuous activities were at higher risk of preterm delivery [6].

In addition, due to gender social norms, matters to do with reproductive care are often regarded as women's agenda by most communities, especially those with misconceptions or lack of knowledge of male involvement in maternal health. As a result, some men's lack of support (even financial support where required) during maternal healthcare seeking may be due to a belief that men are not responsible for or should not be involved in issues related to maternal health [15].

In many African societies, including Tanzania, men are considered breadwinners and expected to bring in income to support the family and provide funds for health care [9]. However, men acknowledged poverty as a primary challenge to their inability to fulfill this role and provide needed maternal health and healthcare supplies. This demonstrates how social norms intersect with the division of labour to influence access to resources [14].

### Access to resources in relation to the utilization of reproductive health

On the other hand, this study found that across all ethnic groups in the study area, women and girls do not have equal access to or control over resources. Men had more access to and control over resources within the community and household levels. Lack of access to and control over resources affects women's reproductive health service utilization. This finding concurred with the previous qualitative study conducted in Uganda [15], which showed that the timing and the type of reproductive care women received depended on their husband's financial support. This can be explained by the fact that when women rely on their husbands for financial support, it may result in delayed access to or limited utilization of reproductive health services [16].

Women may not go to health facilities because they lack money for their fares or to pay for their medicine. The 2022 TDHS - MIS Tanzania household survey report showed that 21.7% of women in Simiyu could not seek treatment for their children with fever in health facilities because of a lack of money [17]. Likewise, the survey also showed that only 36.0% attended four or more ANC, which was the lowest in the Tanzania mainland. This is partly due to the lack of resources where women cannot afford even the fare to reach the health facilities. This shows how the control of resources by men impacts women's health. Even when women are economically empowered, negative attitudes and values still prevent them from having control over those resources, limiting them from utilizing reproductive health [16].

### Gender Roles and responsibilities in relation to the utilization of reproductive health

Some participants in this study affirmed that women and girls have a more significant burden of roles and responsibilities at the household and community level than men and boys. However, men's support for their wives or partners during pregnancy is crucial. Supportive roles and responsibilities are needed, including caring for the children, fetching firewood, farming, harvesting, taking women to the hospital, providing money for healthcare costs, and cooking for them. A previous study conducted in Tanzania showed that male involvement in ANC was significantly associated with attendance at four or more ANC visits. This could be due to the fact that women who went to RMNCAH with their spouses received health education together. This implies that educating a woman with her spouse about the importance of healthcare for the family could potentially promote health-seeking behavior, including ANC [18]. Another study in Nigeria has shown men's involvement during pregnancy has been shown to positively impact maternal health behavior, such as access to and utilization of reproductive health care [19]. A study in Malawi showed that when men played active roles in maternal health, they were better informed and aware of pregnancy-related risks, encouraged women to attend ANC visits (and accompanied them), and encouraged them to meet their dietary requirements [20]. In addition, they provided emotional support and other necessities during delivery. The study reported that when men took up these otherwise non-conventional gender roles, it improved their attitudes toward fatherhood.

## Conclusions

In Tanzania, reducing maternal mortality has been given a high priority. It is addressed in various national commitments, including Tanzania Vision 2025 [21], the National Strategy for Growth and Reduction of Poverty (NSGRP) [22], and the Health Sector Strategic Plan V [23]. Likewise, globally, an urgent need for an intensive effort has been stressed to impede the fulfillment of Sustainable Development Goals (SDGs), especially SDG 3 and 4.

Based on the findings and commitment mentioned above, the paper explore gender-based enablers and/or barriers that influence women and girls' realization of their sexual and reproductive health and rights in the Simiyu Region of Tanzania. It was found that social norms, decision-making powers, and lack of access and control over resources impact women's utilization of quality RMNCAH services like family planning. Women's roles of taking care of the families have tended to limit their time to access the required services. However, changes are now observed with continuous community sensitization and increased scope of women's participation in decision-making at the family and community levels. Interventions to support women's decision-making are essential to improving gender equality in RMNCAH services and utilization in Simiyu, Tanzania. This paper provides opportunities to transform how we work in a manner where difference is valued and celebrated for a prosperous, sustainable future for all and overcome gender inequities that influence woman's use of RMNCAH services in Tanzania.

## Data Availability

All data sets are available on request to the corresponding author and the organization at large.

## Abbreviations

AMO: Assistant Medical Officer
CEDAW: Convention on the Elimination of All Forms of Discrimination Against Women
CHW: Community Health Care Worker
DMO: District Medical Officer
FBOs: Faith-based organizations
FGDs: Focus Group Discussions
F.P.: Family Planning
GAC: Global Affairs Canada
GBV: Gender-based violence
GII: Gender Inequality Index
GNA: Gender Needs Assessment
IPV: Intimate partner violence
K.I.s: Key Informants
MoHCDGEC: Ministry of Health, Community Development, Gender, Elderly, and Children
NBS: National Bureau of Statistics
NPA-VAWC: National Plans of Action to End Violence Against Women and Children
NSGRP: National Strategy for Growth and Reduction of Poverty
PO-RALG: President's Office – Regional Administration and Local Government
RMNCAH: Reproductive, Maternal, New-born, Child, and Adolescent Health
SDGs: Sustainable Development Goals
S.P.: Service Provider
SRH: Sexual Reproductive Health
SRHR: Sexual and Reproductive Health and Rights
TDHS-MIS: Tanzania Demographic Health Survey and Malaria Indicator Survey
WASH: Water, Sanitation, and Hygiene
WHO: World Health Organization
SSA: Sub-Saharan Africa
